# Detection of LINE-1 hypomethylation in cfDNA of Esophageal Adenocarcinoma Patients

**DOI:** 10.3390/ijms21041547

**Published:** 2020-02-24

**Authors:** Elisa Boldrin, Matteo Curtarello, Marco Dallan, Rita Alfieri, Stefano Realdon, Matteo Fassan, Daniela Saggioro

**Affiliations:** 1 Immunology and Molecular Oncology Unit, Istituto Oncologico Veneto IOV-IRCCS, Via Gattamelata 64, 35128 Padova, Italy; matteo.curtarello@iov.veneto.it (M.C.); marcoblock.85@libero.it (M.D.); daniela.saggioro@iov.veneto.it (D.S.); 2 Oncological Surgery, Veneto Institute of Oncology IOV-IRCCS, via dei Carpani 16, 31033 Castelfranco Veneto, Italy; rita.alfieri@iov.veneto.it; 3 Endoscopy Unit, Veneto Institute of Oncology IOV-IRCCS, via Gattamelata 64, 35128 Padova, Italy; stefano.realdon@iov.veneto.it; 4 Department of Medicine (DIMED), Surgical Pathology and Cytopathology, University of Padova, via Giustiniani 2, 35128 Padova, Italy; matteo.fassan@gmail.com

**Keywords:** LINE-1 hypomethylation, Barrett’s esophagus (BE), Esophageal adenocarcinoma (EADC), cfDNA, liquid biopsy

## Abstract

DNA methylation plays an important role in cancer development. Cancer cells exhibit two types of DNA methylation alteration: site-specific hypermethylation at promoter of oncosuppressor genes and global DNA hypomethylation. This study evaluated the methylation patterns of long interspersed nuclear element (LINE-1) sequences which, due to their relative abundance in the genome, are considered a good surrogate indicator of global DNA methylation. LINE-1 methylation status was investigated in the cell-free DNA (cfDNA) of 21 patients, 19 with esophageal adenocarcinoma (EADC) and 2 with Barrett’s esophagus (BE). The two BE patients and one EADC patient were also analyzed longitudinally. Methylation status was analyzed using restriction enzymes and DNA amplification. This methodology was chosen to avoid bisulfite conversion, which we considered inadequate for cfDNA analysis. Indeed, cfDNA is characterized by poor quality and low concentration, and bisulfite conversion might worsen these conditions. Results showed that hypomethylated LINE-1 sequences are present in EADC cfDNA. Furthermore, longitudinal studies in BE suggested a correlation between methylation status of LINE-1 sequences in cfDNA and progression to EADC. In conclusion, our study indicated the feasibility of our methodological approach to detect hypomethylation events in cfDNA from EADC patients, and suggests LINE-1 methylation analysis as a new possible molecular assay to integrate into patient monitoring.

## 1. Introduction

Esophageal adenocarcinoma (EADC) is a highly lethal malignancy [1,2,3], the incidence of which is continually increasing in Western countries [4,5]. Unfortunately, despite implementation of the latest therapeutic approaches, patient outcomes remain generally unfavorable [2], and no indicators that allow monitoring of the duration of therapy response or early detection of recurrence are available yet. Thus, it is of particular relevance to improve the understanding of the molecular basis of the disease and to develop new monitoring tools.

EADC genetics is characterized not by specific gene mutations, by a strong genomic instability that leads to complex alterations, including gains and losses of numerous chromosomal regions and gene copy number variations [6]. However, besides genetic alterations, epigenetic changes have also been described with particular relevance to variation in DNA methylation [7,8,9].

Genome-wide methylation analysis conducted in a variety of neoplasias has revealed that, next to a selective hypermethylation at the CpG islands of specific tumor suppressor gene promoters, the dominant epigenetic change is global hypomethylation [10,11,12]. This phenomenon is particularly evident in the repetitive DNA sequences that account for about a half of the human genome. Long interspersed nuclear element 1 (LINE-1) sequences are one type of repetitive sequence dispersed throughout the genome. They represent almost 17% of the entire genome. Their hypomethylation has been frequently reported as a common epigenetic event in different malignancies and, for this reason, they have been indicated as possible surrogate markers of the methylation status of the entire genome [13]. DNA methylation within the promoter region of human LINE-1 elements is important for maintaining transcriptional inactivation, for inhibiting transposition, and consequently for contributing to genome stability [14,15].

In EADC, both the genetic and epigenetic alterations are probably the consequence of chronic inflammation associated with gastro-esophageal reflux, obesity, and smoking, which are important risk factors [7,16]. Sustained inflammation has also been listed among the conditions that promote the release of circulating cell-free DNA (cfDNA) in blood [17].

In previous studies, we showed that in the cfDNA of dysplastic Barrett’s esophagus or EADC patients it was possible to detect tumor-related genetic alterations such as loss of heterozygosity (LOH), and that this alteration could be used to monitor patient outcomes [18,19].

In both esophageal cancer subtypes, squamous cell carcinoma (ESCC) and adenocarcinoma (EADC), DNA methylation status has been widely studied in terms of hypermethylation at the single oncosuppressor gene promoter level [9,20,21,22,23,24,25]. In contrast, whole-genome hypomethylation using LINE-1 sequences has been principally investigated in ESCC [26,27,28,29] and, to our knowledge, no data on LINE-1 status in EADC and its possible use as surrogate marker are so far available. However, a recent study demonstrated that LINE-1 retrotransposition is active in EADC and in the precursor condition Barrett’s esophagus (BE) [30]. These data suggest that the LINE-1 sequences could also be hypomethylated and contribute to genomic instability in EADC.

In this study, we investigated the presence of hypomethylated LINE-1 sequences in the cfDNA of EADC patients. LINE-1 sequences were chosen since their abundance and distribution throughout the genome indicate them as a good biomarker with which to detect hypomethylation of circulating tumor-derived DNA, usually present at low levels in the bloodstream. DNA methylation analysis is based on three major approaches: (i) bisulfite conversion of methylated cytosine in CpG islands, (ii) 5′-methylcytosine immunoprecipitation, and (iii) digestion with methylation-sensitive restriction enzymes. We chose the methylation-sensitive restriction approach [31,32], which we considered less challenging than bisulfite conversion for cfDNA analysis.

## 2. Results and Discussion

### 2.1. LINE-1 Methylation Analysis in Formalin Fixed Paraffin Embedded (FFPE)-DNA

**Figure 1:**
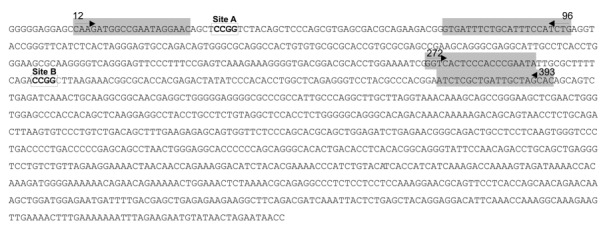
Long interspersed nuclear element 1 (LINE-1) promoter sequence. Recognition sites of MspI/HpaII isoschizomers are shown in bold; grey boxes and arrows indicate forward and reverse primers.

Before analyzing the LINE-1 methylation status in the cfDNA of EADC patients, we tested the efficiency of the restriction enzyme approach in a few FFPE tumor DNA samples. We digested DNA with the methylation-sensitive isoschizomers MspI and HpaII; after digestion, we amplified using real-time PCR (rtPCR) the LINE-1 promoter region between Nucleotides 272 and 393, which includes at Nucleotides 305-308 one recognition site (CCGG) for the enzymes (Figure 1).

**Figure 2:**
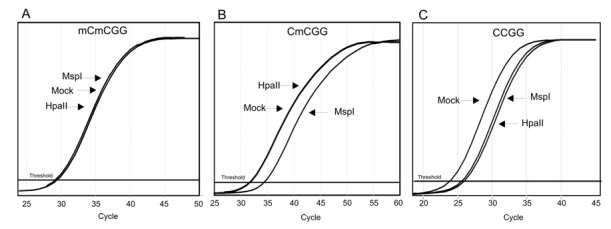
Real-time amplification curves of digestion products. The profiles indicate the methylation status of the two cytosine residues present at the restriction site: (**A**) both cytosines methylated; (**B**) only internal cytosine methylated; (**C**) both cytosines unmethylated.

Both cytosines (CC) at the recognition site could be methylated, thus giving rise to different digestion patterns [33]. MspI cuts CCGG and CmCGG, but not when the external cytosine is methylated (mCCGG and mCmCGG), while HpaII cuts only unmethylated CCGG and, with a lower efficiency, mCCGG. These different cutting abilities gave rise to different amplification curves, since only undigested DNA was amplified (Figure 2).

Residual methylation (LINE-1 methylation level (%)) was given by the difference in cycle threshold (Ct) of the amplification curves (ΔCt = Ct of digested sample – Ct of mock) and was calculated using the following formula: 2^−ΔCt^ × 100.

**Figure 3:**
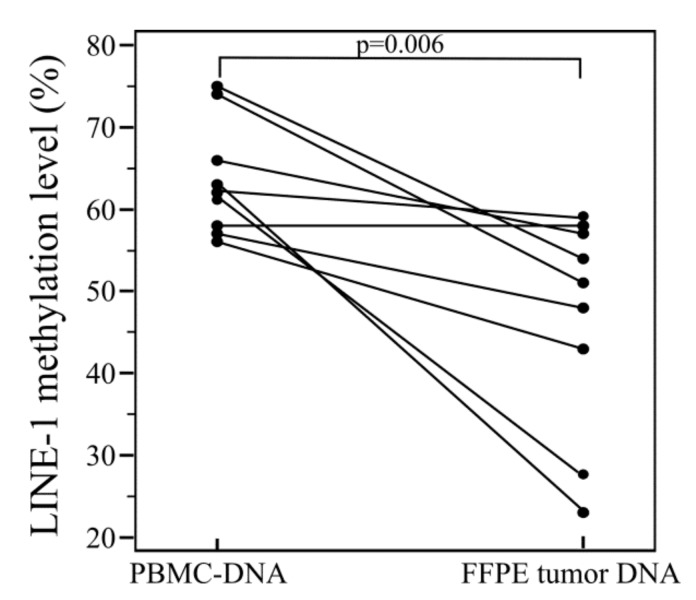
Comparison of LINE-1 methylation levels between constitutive DNA and FFPE tumor DNA. *p*-values were calculated using *t*-test.

Using this approach, we were able to detect the presence of hypomethylated LINE-1 sequences in tumor FFPE-DNA. Furthermore, the difference between constitutive (i.e., PBMC-DNA) and tumor DNA was statistically significant (*p* = 0.006) (Figure 3).

These data suggest that LINE-1 hypomethylation could be used as a surrogate marker in EADC samples, as suggested for ESCC subtype [26,28]. Encouraged by these results, we then extended the analysis to the cfDNA of EADC patients.

### 2.2. LINE-1 Methylation Analysis in cfDNA

For this analysis, we tested the methylation level of two CCGG sequences located in the LINE-1 promoter, one at Nucleotides 36–39 and one at Nucleotides 305–308, already used in FFPE analysis (defined as Site A and site B throughout the manuscript) (Figure 1).

**Figure 4:**
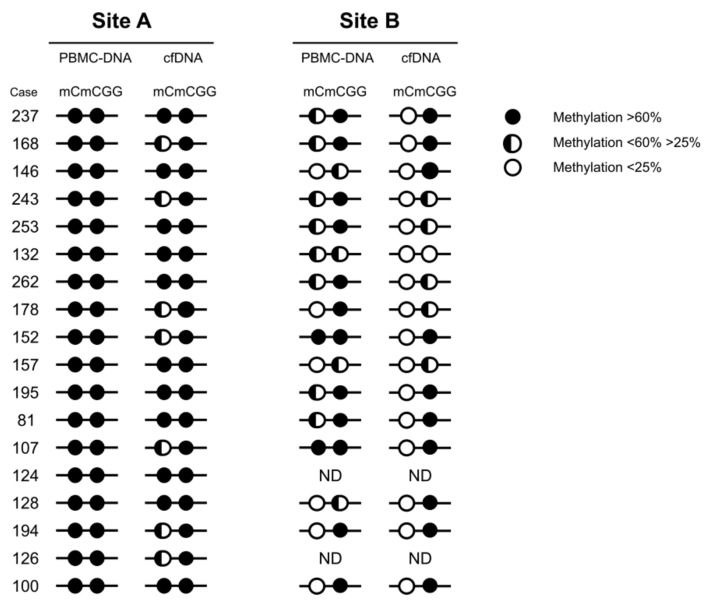
A schematic representation comparing the LINE-1 methylation level between PBMC-DNA and cell-free DNA (cfDNA) of esophageal adenocarcinoma (EADC) patients. Each dot represents one cytosine residue.

**Figure 5:**
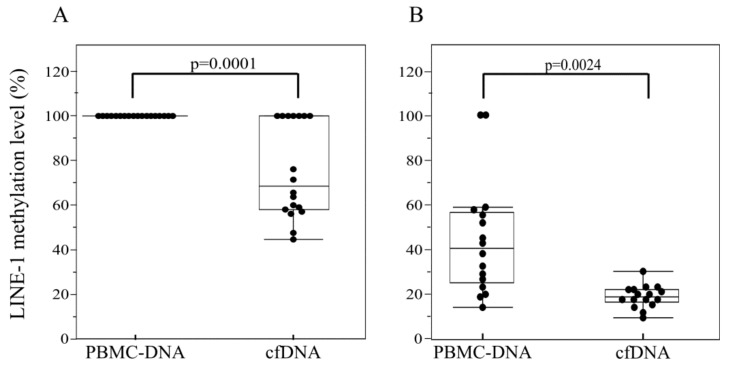
Dot plot of LINE-1 methylation level. Status of external cytosine of site A (**A**) and of site B (**B**) in PBMC and cfDNA.

The methylation status of all four cytosines is schematically represented in Figure 4. Regarding Site A, we found that the internal C was always heavily methylated in both the PBMC-DNA and cfDNA of all cases (Figure 4). In contrast, the external one, which was fully methylated in PBMC-DNA, showed a statistically significant demethylation pattern in cfDNA (Figure 4 and Figure 5A). Indeed, over 60% (11/18) of patients had a partial hypomethylation in their cfDNA, with a median methylation level of 67% vs. 100% of constitutive DNA (*p* = 0.0001) (Figure 5A).

In Site B, the external cytosine residue, although partially demethylated in constitutive DNA, showed a very low residual methylation level, ranging from 6% to 24%, in cfDNA samples, with a median methylation level of 14% vs. 33% (*p* = 0.0024; Figure 5B). In contrast, no significant difference in methylation of the internal cytosine residue of site B was found between PBMC-DNA and cfDNA (Figure 4).

These results showed that this approach, based on a restriction enzyme digestion and DNA amplification, allowed the detection of hypomethylated LINE-1 sequences in cfDNA of EADC patients.

In several types of tumors, including ESCC [28,29], LINE-1 hypomethylation has been found to be associated with a poor prognosis [34,35,36]. Unfortunately, due to the limited number of patients examined, we were unable to determine any correlation between hypomethylation status and patient outcome. Further studies with a larger cohort need to be carried out to clarify this point.

### 2.3. LINE-1 Methylation Analysis in cfDNA of Longitudinal Cases

Inspired by the possibility of using LINE-1 methylation status to predict tumor behavior, we extended the analysis to a few longitudinal cases, one EADC and two BE; one BE patient progressed to low-grade dysplasia (LGD) and high-grade dysplasia (HGD)/EADC during surveillance, while the other remained stable.

**Figure 6:**
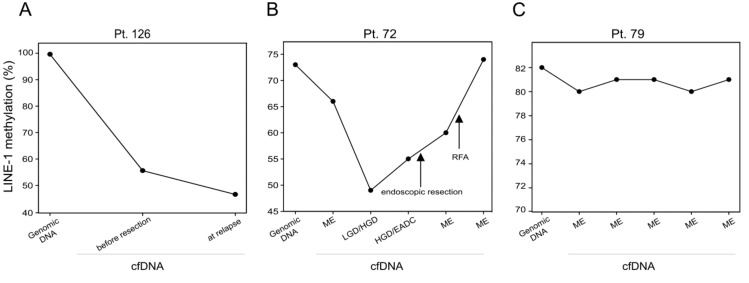
LINE-1 methylation level in longitudinal studies of a EADC patient (**A**), of a Barrett’s esophagus (BE) progressor patient (**B**) and of a stable metaplastic BE patient (**C**).

**Patient 126.** (76 years old) The patient was diagnosed with a Stage T2N0M0 EADC. Analysis revealed that compared to the constitutive DNA, the methylation level of LINE-1 was lower in a cfDNA sample obtained at the time of resection; LINE-1 hypomethylation was also present and at an even higher level in the cfDNA gathered one year later when recurrence was recorded (Figure 6A).

**Patient 72.** (69 years old) The patient was included in a BE monitoring program at our institution. The patient presented an increased LINE-1 sequence hypomethylation during the progression from metaplastic epithelium (ME) to HGD/EADC. However, after endoscopic resection and successive radio frequency ablation (RFA), LINE-1 methylation level increased and switched back to the level of constitutive DNA. Histological examination revealed the absence of HGD/EADC and the presence of a metaplastic epithelium (Figure 6B).

**Patient 79.** (75 years old) This patient also had BE and participated in the monitoring program of our institution. Blood samples were collected during four years of follow-up; the patient did not show any dysplastic occurrence during this period. In his cfDNA, no significant variations in methylation status of LINE-1 promoter sequences were detected (Figure 6C).

Taken together, these longitudinal analyses suggest that LINE-1 methylation could be used to follow EADC patient outcomes. More interestingly, the data obtained in BE patients indicated that LINE-1 hypomethylation analysis could be used to monitor the EADC pre-neoplastic condition.

These data also suggest that global DNA hypomethylation, of which LINE-1 is a surrogate marker, might be associated with the initiation of the neoplastic process.

## 3. Materials and Methods

### 3.1. Patients

The study was carried out according to the Code of Ethics of the World Medical Association (Declaration of Helsinki and its later amendments), and had the approval of the Ethics Committee for Clinical Trials (cod. number CE IOV 2012/65 of 26 February 2018). A total of 21 patients, 19 with EADC and 2 with Barrett’s esophagus (BE), were included in this exploratory study. Two BE patients and one EADC patient were studied longitudinally. The EADC median age at diagnosis was 67 (range 44–77); all patients except one were male, 77% had stage III tumor, 11% stage I, and 6% stage II or IV. All patients gave written informed consent in accordance with the Helsinki Declaration.

### 3.2. DNA Extraction

Plasma was isolated from corpuscular components of the blood by two subsequent centrifugations (2000 *g* and 16000 *g*, respectively) within 2 h of blood collection, and was stored at −80 °C until cfDNA extraction. One aliquot of whole blood was also stored for germline DNA extraction, which was used as a reference. cfDNA was extracted from 1 mL of plasma using the QIAamp Circulating Nucleic Acid Kit (Qiagen, Milan, Italy); germline DNA was isolated using the automated extractor MagNA Pure Compact Instrument (Roche, Milan, Italy), and a QIAamp Mini Kit (Qiagen, Milan, Italy) was used to isolate DNA from FFPE tumor samples. Only FFPE with a neoplastic component ≥70% was considered adequate for tumor DNA analysis; otherwise, when necessary, samples were enriched by manual macro-dissection of eight consecutive 10 µm thick sections. DNA quantity was assessed with a NanoDrop 1000 spectrophotometer (Thermo Fisher Scientific, Monza, Italy). cfDNA quality was further evaluated with the Agilent 2100 Bioanalyzer using the High Sensitivity DNA kit (Agilent Technologies, Milan, Italy).

### 3.3. Methylation-Sensitive Restriction Enzyme Digestion and rtPCR

To assess the methylation status of LINE-1 repetitive elements, we investigated two regions within the LINE-1 promoter (GenBank accession number X58075.1), one at Position 12-96 and one at Position 272-393 (Figure 1). The two regions, defined respectively as “Site A” and “Site B” throughout the paper, contain the CCGG recognition site of the two isoschizomers MspI and HpaII, the cleavage capability of which depends on the methylation pattern of the two cytosines present at the recognition site. Indeed, MspI can cleave CCGG and CmCGG and it is inhibited by the methylation of the external cytosine (mCmCGG). HpaII can cleave CCGG and, with less efficiency, mCCGG, and it is inhibited by the methylation of the internal cytosine (CmCGG). A total 125 ng of DNA was digested in a final volume of 10 ul using MspI and HpaII (New England BioLabs, Ipswich, MA, USA). To minimize pipetting errors during sample preparation for DNA digestion, we prepared a reaction master mix including DNA and the Smart-cut buffer (New England BioLabs, Ipswich, MA, USA) for the three reactions (mock and MspI or HpaII digestion). The mixture was mixed thoroughly and 10 uL dispensed into three tubes; a measure of 1 uL of nuclease-free water was added to mock tube and 1 uL of MspI or HpaII was added to the remaining tubes. The digestion reaction was performed at 37 °C for 2.5 h, and enzymes inactivated at 80 °C for 20′. Digested DNA was then amplified by rtPCR technique. Primer sequences were CAAGATGGCCGAATAGGAAC (FW) and CAGATGGAAATGCAGAAATCAC (RW) for Site A, and GGTCACTCCCACCCGAATA (FW) and GTGCTAGCAATCAGCGAGAT (RW) for Site B. rtPCR was performed using a Rotor Gene Q thermocycler (Qiagen, Milan, Italy) using the following conditions: 95 °C for 3′ and then 60 cycles of 95 °C for 20′’, 58 °C for 20′,’ and 72 °C for 30′’, with a final extension of 72 °C for 5′. Green dye (Diatech Pharmacogenetics, Jesi, Italy) was used as the intercalant dye to detect PCR products. Ideally, if one or both cytosines were fully demethylated, no amplification product would be detected for the corresponding enzyme or both, due to complete cleavage of the template. However, due to the rarity of 100% demethylation and to the possible existence of LINE-1 variants with mutations in the CCGG sequence, it was always possible to detect amplicons.

To calculate the methylation level of the external C of each site, we used the formula 2^−ΔCt^ × 100, where the ΔCt was the difference between the Ct value of the MspI digested sample and the Ct value of the undigested sample (mock). Similarly, to calculate the methylation level of the internal C of each site, we used the same formula, where the ΔCt was the difference between the Ct value of the HpaII digested sample and the Ct value of the undigested sample.

### 3.4. Statistics

All statistical analyses were performed using MedCalc version 12.2.1 (MedCalc Software, Ostend, Belgium). A paired *t*-test was performed to compare LINE-1 methylation levels between groups. All statistical tests were two-sided and *p* values ≤ 0.05 were considered significant.

## 4. Conclusions

In this study, we assessed the potential applicability of a methylation-sensitive restriction enzyme approach to investigate LINE-1 methylation status in cfDNA of EADC patients. Results indicated that the hypomethylation status of LINE-1 could be used as a tumor surrogate marker not only to monitor EADC patients, but also as an additional tool in BE patient surveillance.
